# Proximal Tubular Hypertrophy and Enlarged Glomerular and Proximal Tubular Urinary Space in Obese Subjects with Proteinuria

**DOI:** 10.1371/journal.pone.0075547

**Published:** 2013-09-25

**Authors:** Ana Tobar, Yaacov Ori, Sydney Benchetrit, Gai Milo, Michal Herman-Edelstein, Boris Zingerman, Netta Lev, Uzi Gafter, Avry Chagnac

**Affiliations:** 1 Department of Pathology, Rabin Medical Center, Petah Tikva, Israel; 2 Department of Nephrology & Hypertension, Rabin Medical Center, Petah Tikva, Israel; 3 Department of Nephrology, Meir Medical Center, Kfar Saba, Israel; 4 Sackler School of Medicine, Tel Aviv University, Tel Aviv, Israel; University College London, United Kingdom

## Abstract

**Background:**

Obesity is associated with glomerular hyperfiltration, increased proximal tubular sodium reabsorption, glomerular enlargement and renal hypertrophy. A single experimental study reported an increased glomerular urinary space in obese dogs. Whether proximal tubular volume is increased in obese subjects and whether their glomerular and tubular urinary spaces are enlarged is unknown.

**Objective:**

To determine whether proximal tubules and glomerular and tubular urinary space are enlarged in obese subjects with proteinuria and glomerular hyperfiltration.

**Methods:**

Kidney biopsies from 11 non-diabetic obese with proteinuria and 14 non-diabetic lean patients with a creatinine clearance above 50 ml/min and with mild or no interstitial fibrosis were retrospectively analyzed using morphometric methods. The cross-sectional area of the proximal tubular epithelium and lumen, the volume of the glomerular tuft and of Bowman’s space and the nuclei number per tubular profile were estimated.

**Results:**

Creatinine clearance was higher in the obese than in the lean group (P=0.03). Proteinuria was similarly increased in both groups. Compared to the lean group, the obese group displayed a 104% higher glomerular tuft volume (P=0.001), a 94% higher Bowman’s space volume (P=0.003), a 33% higher cross-sectional area of the proximal tubular epithelium (P=0.02) and a 54% higher cross-sectional area of the proximal tubular lumen (P=0.01). The nuclei number per proximal tubular profile was similar in both groups, suggesting that the increase in tubular volume is due to hypertrophy and not to hyperplasia.

**Conclusions:**

Obesity-related glomerular hyperfiltration is associated with proximal tubular epithelial hypertrophy and increased glomerular and tubular urinary space volume in subjects with proteinuria. The expanded glomerular and urinary space is probably a direct consequence of glomerular hyperfiltration. These effects may be involved in the pathogenesis of obesity-related renal disease.

## Introduction

Obesity is an independent risk factor for chronic kidney disease and for the need for renal replacement therapy. Obesity-related glomerulopathy is a well-defined entity, characterized by glomerulomegaly with or without focal segmental glomerulosclerosis [1]. However, obesity also accelerates the progression of kidney diseases that are not primarily related to obesity, such as IgA nephropathy [2,3], reduced renal mass [4] and possibly renal transplant nephropathy [5]. Obesity and overweight are associated with increased glomerular filtration rate, renal plasma flow and/or filtration fraction [6-14]. The improvement of these renal hemodynamic abnormalities following weight loss [15] supports a cause-and-effect relationship between adiposity and glomerular hyperfiltration. Obesity-related hyperfiltration, associated with increased glomerular hydrostatic pressure [10] and renal hypertrophy are considered significant factors in the pathogenesis of chronic kidney disease [16-20].

Glomerulomegaly is the structural correlate of glomerular hyperfiltration, a finding observed, both in obesity [1,21-31] and diabetes mellitus [32]. Studies in diabetes [33,34] demonstrated that glomerular enlargement is associated with hypertrophy of tubules. Experimental data in animal models suggested that in the diabetic kidney, proximal tubular hypertrophy and increased proximal tubular sodium reabsorption play a central role in the pathogenesis of glomerular hyperfiltration [34]. Glomerular hyperfiltration in obese subjects is associated with increased sodium reabsorption by the proximal tubule [35,13]. No data are available regarding tubular hypertrophy in the obese kidney, neither in humans nor in animal models. Another structural renal abnormality that has been described in a model of obese dogs is an increase in Bowman’s space area [31]. This increase, a possible consequence of glomerular hyperfiltration, has not been reported in obese humans.

The aims of the present study were to determine whether Bowman’s space volume and proximal tubular volume are increased in obese subjects with proteinuria, and if so, whether the latter is due to epithelial hypertrophy, epithelial hyperplasia and/or luminal dilatation. We showed that obesity is associated with proximal tubular epithelial hypertrophy and increased glomerular and tubular urinary space volume in subjects with proteinuria.

## Materials and Methods

### Ethics Statement

The protocol of this study was approved by the Institutional Review Board of the Rabin and Meir Medical Centers. Informed consent was not required as this was a retrospective study. The Institutional Review Boards specifically waived the need for informed consent.

We searched the Rabin Medical Center and Meir Medical Center database to identify patients who had undergone native kidney biopsies, for whom data were available regarding body mass index (BMI) at the time of biopsy and who matched the following inclusion and exclusion criteria. Patients were included if they were ≥ 17 years of age; had a BMI below 25 or a BMI of 30 or higher; were non-diabetic, i.e. had, in the absence of anti-diabetic medications, a fasting blood glucose below 100 mg/dl or a fasting blood glucose above 99 mg/dl and below 126 mg/dl with a Hba1c level below 6.5% [36]; had a creatinine clearance of 50 ml/min or higher. Patients were excluded if: they had overt diabetes mellitus or if they received anti-diabetic medications for impaired glucose tolerance; they had moderate to severe heart failure, reduced renal mass from any cause (unilateral agenesis, dysplastic kidney, s/p partial or unilateral nephrectomy), renovascular disease, obstructive uropathy, upper urinary tract infection, renal malignancy or a history of malignant hypertension or acute kidney injury. Patients were excluded if the kidney biopsy showed glomerular endocapillary proliferative features, crescents, a membranoproliferative pattern, necrotizing or exudative lesions, thrombotic microangiopathy and moderate to severe glomerulosclerosis. In addition, cases with moderate to severe interstitial fibrosis, tubulointerstitial disease, deposition diseases as amyloidosis, fibrillary glomerulonephritis and hereditary metabolic diseases were excluded.

### Assessment of glomerular and tubular size

Using a digital camera and PAS-stained sections, we captured at x400 magnification 15 light microscopic fields containing consecutively encountered proximal tubules. We captured at x200 magnification all light microscopic fields available from the biopsy material containing non-globally sclerotic glomerular profiles. The images were coded. Using Adobe Photoshop software, a stereological grid consisting of uniformly spaced points was superimposed over each picture. The grid’s points were spaced by 27.8 μ for the glomeruli and 13.9 μ for the tubules.

A single blinded examiner (A.T.) obtained the following parameters using the point counting principle:

For glomerular profiles:
  - N of points over the glomerular tuft
  - N of points over Bowman space

For proximal tubular profiles:
  - N of points over the proximal tubular profile (cells, including brush border), excluding the lumen
  - N of points over the lumen of the proximal tubular profile
  - N of nuclei in the proximal tubular profile

### Calculations

The surface area of each of the glomerular and tubular profiles was calculated as: A = d^2^ x N, where A is the surface area of the measured profile, d is the distance between 2 points on the grid and N is the number of points over the measured profile. The cross sectional surface area was estimated for the glomerular tuft (A_tuft_), Bowman space (A_BS_), proximal tubular cells (A_PTC_) and proximal tubular lumen (A_PTL_) respectively. The cross sectional surface area of the glomeruli (A_glom_) was estimated as A_tuft_ + A_BS_. Mean glomerular tuft volume (V_tuft_) was calculated according to Weibel [37]: V_tuft_= (β/κ)·(A_tuft_)^3/2^, where β = 1.38 is the shape coefficient for spheres, κ = 1.1 is the size distribution coefficient, and A is the glomerular tuft cross-sectional area. Mean glomerular volume (tuft and Bowman space volume all together) (V_glom_) was calculated as V_glom_ = (β/k)x (A_tuft_ + A_BS_)^3/2^. The volume of Bowman space (V_BS_) was calculated as V_Bowman_'_s space_ = V_glom_- V_tuft_. The glomerular tuft area, glomerular Bowman space area, proximal tubular cells area and proximal tubular lumen area for each biopsy were calculated using the median area of the respective structures.

Creatinine clearance (measured creatinine clearance) was calculated from creatinine measurements in 24-hour urine collections and in serum in 10 out of the 11 obese subjects and in 11 out of the 14 lean subjects. Measured creatinine clearance (ml/min) = 24-hour creatinine excretion (mg) / serum creatinine (mg/ml) x 1440 (min). In order to estimate creatinine clearance for the whole population (14 lean and 11 obese subjects), we calculated creatinine clearance using the Cockroft-Gault equation: creatinine clearance (ml/min) = (140 – age (year)) x body weight (kg) x (0.85 if female) / 72 x serum creatinine (mg/dl).

### Statistics

Normally distributed data are expressed as mean ± SD. Variables with skewed distribution are expressed as median (25-75 percentiles). The significance of differences between the groups was evaluated by a two-tailed Student’s t-test for normally distributed data and by a Mann-Whitney U test for non-normally distributed data. The Pearson’s chi-square test was used to compare the gender distribution between the groups. The analyses were carried out using SPSS version 17.0.

## Results

### Population characteristics

Table 1 shows the characteristics of the studied population. Fourteen lean and 11 obese Caucasians subjects were studied. Gender distribution was similar. The obese group was older by 16 years, the age range varying between 17 and 60 for the lean subjects and 30 to 67 for the obese. BMI was 17 points higher in the obese group compared to the lean one, ranging from 16.8 to 24.8 and from 33.6 to 53.4 in the lean and obese groups, respectively. Height was similar. Systolic blood pressure was higher in the obese group; diastolic blood pressure was similar in both groups. Serum fasting blood glucose, non-significantly higher in the obese group, was normal in both groups. Fasting blood glucose ranged from 4.3 to 5.4 mmol/L in the lean group and from 4.3 to 6.6 mmol/L in the obese group. Hba1c was 5.8±0.4% (normal value range: 4.0 to 6.1%) in the obese group, ranging from 5.2 to 6.3% (n=8), reflecting the fact that a proportion of these subjects had impaired glucose tolerance. For 3 subjects, no data about Hba1c at the time of the biopsy were available. Their fasting blood glucose was 4.3, 4.6 and 4.7 mmol/L. Serum creatinine and urea were similar in both groups. Estimated creatinine clearance was 44% higher in the obese group, and measured creatinine clearance was 49% higher, compared to the lean group. Proteinuria was similarly increased in both groups. Serum albumin was similar in both groups. The histopathological diagnoses in the control group were: focal segmental glomerulosclerosis (n=4), diffuse mesangial proliferation (n=1), IgA nephropathy (n=3), membranous nephropathy (n=2), thin basement membrane disease (n=1), nonspecific abnormalities (n=1) and no abnormalities (n=2). The histopathological diagnoses in the obese group were obesity-related glomerulopathy (n=9), IgA nephropathy (n=1) and nonspecific abnormalities (n=1).

**Table 1:** Population characteristics.

	**Lean group (n=14**)	**Obese group (n=11**)	**P**
**Age (yrs**)	30±12	46±10	0.002
**Gender (m/f**)	7/7	5/6	0.8
**Height (m**)	1.69±0.10	1.70±0.13	0.8
**Body weight (kg**)	61±11	115±20	*
**BMI**	21.6 (19.0-23.0)	38.9 (36.5-41.4)	*
**Fasting blood glucose (mmol/L**)	4.9±0.3	5.3±0.7	0.07
**Serum creatinine (μmol/L**)	72 (59-91)	88 (65-111)	0.3
**Serum urea (mmol/L**)	9.3 (6.8-12.1)	12.1 (10.4-15.0)	0.15
**Measured creatinine clearance (mL/min**)**^a^**	97±22	145±62	0.03
**Estimated creatinine clearance (mL/min**)**^b^**	103±27	148±55	0.01
**Serum Albumin (g/L**)	39±7	40±4	0.9
**Proteinuria (g/d**)	1.5 (0.5-1.7)	2.0 (1.2-4.5)	0.13
**Systolic BP (mm Hg**)	119 (115-120)	135 (127-145)	0.02
**Diastolic BP (mm Hg**)	76 (71-80)	80 (79-85)	0.2

* different by definition

^a^ N=11 and 10 for the lean and obese groups, respectively

^b^ Cockcroft-Gault equation

### Structural data

**Figure 1:**
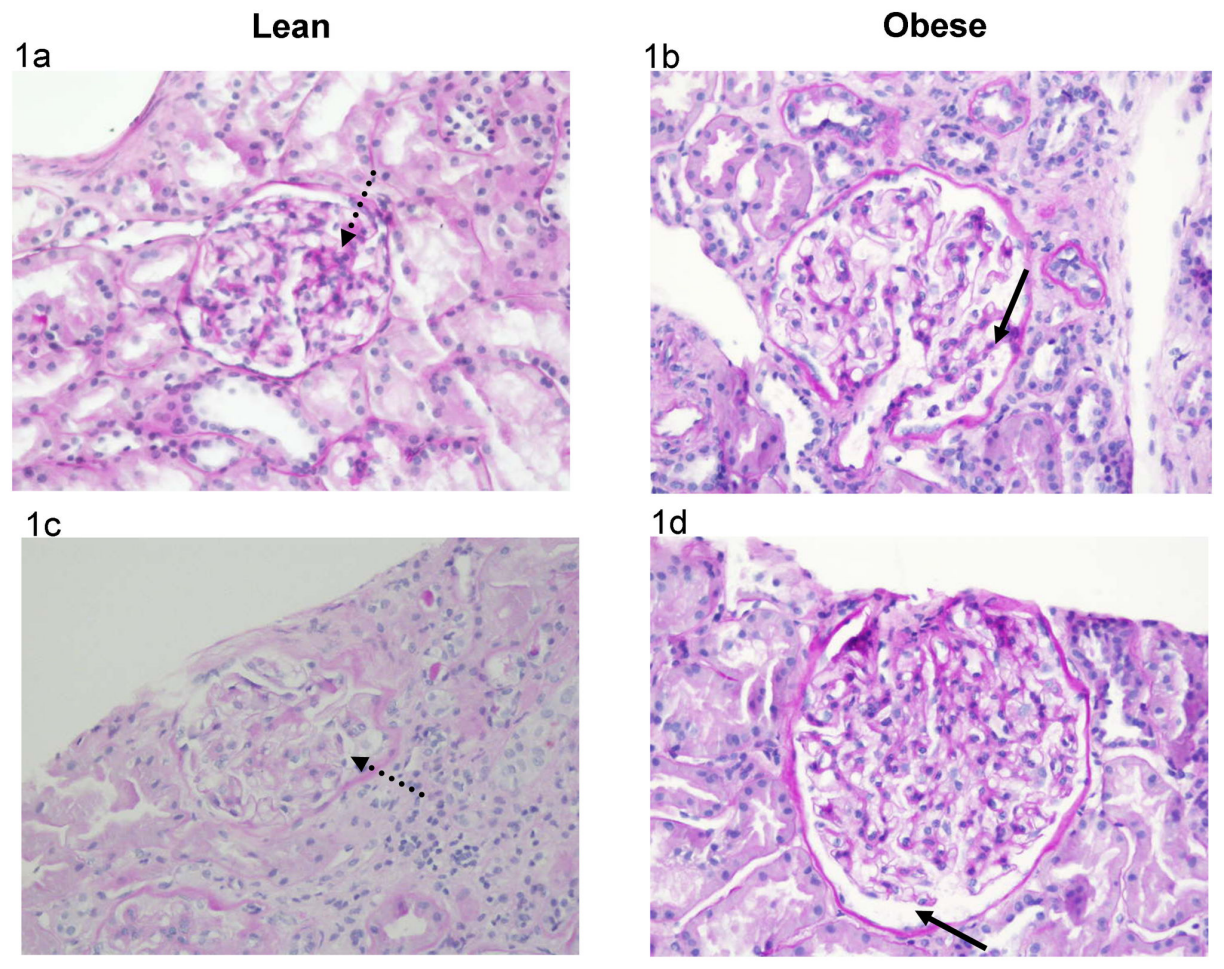
Glomerular Tuft and Bowman’s Space Cross Sectional Area in Lean and Obese Subjects. Figure 1a & 1b: The cross sectional area of the glomerular tuft area is larger in the obese than in the lean subjects (original magnification x200). (a) Lean subject: the cross sectional area of this glomerular tuft (dotted arrow) is 15,460 μ^2^ (mean cross sectional area of the lean group: 15,800 μ^2^). (b) Obese subject: the cross sectional area of this glomerular tuft (arrow) is 24,730 μ^2^ (mean cross sectional area of the obese group: 25,500 μ^2^). Figure 1c & 1d: The cross sectional area of Bowman’s space is larger in the obese than in the lean subjects (original magnification x200). (c) Lean subject: the area of this Bowman’s space cross section (dotted arrow) is 3090 μ^2^ (mean cross sectional area of the lean group: 3400 μ^2^). (d) Obese subject: the area of this Bowman’s space cross section (arrow) is 4640 μ^2^ (mean cross sectional area of the obese group: 4800 μ^2^).

**Figure 2:**
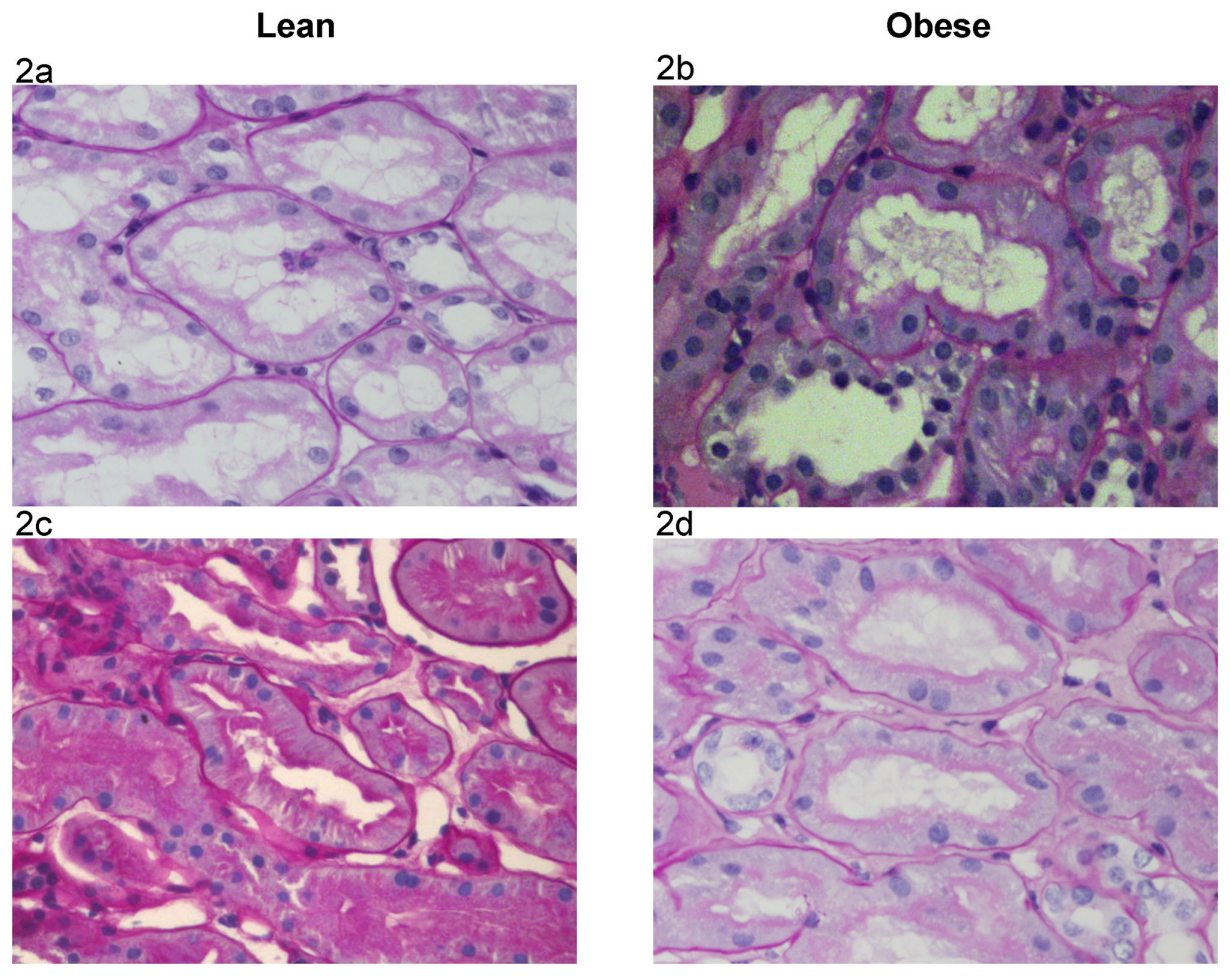
Proximal Tubular Epithelium and Tubular Lumen Cross Sectional Area in Lean and Obese Subjects. Figure 2 a & b: The cross sectional area of the proximal tubular epithelium is larger in the obese than in the lean subjects (original magnification x400). (a) Lean subject: the cross sectional area of this proximal tubular epithelium (dotted arrow) is 2995 μ^2^ (mean cross sectional area of the lean group: 2980 μ^2^). (b) Obese subject: the cross sectional area of this proximal tubular epithelium (arrow) is 4060 μ^2^ (mean cross sectional area of the obese group: 3950 μ^2^). Figure 2 c & d: The cross sectional area of the proximal tubular lumen is larger in the obese than in the lean subjects (original magnification x400). (c) Lean subject: the cross sectional area of this proximal tubular lumen (dotted arrow) is 970 μ^2^ (mean cross sectional area of the lean group: 960 μ^2^). (d) Obese subject: the cross sectional area of this proximal tubular lumen (arrow) is 1550 μ^2^ (mean cross sectional area of the obese group: 1480 μ^2^).

The number of glomerular sections was 20 (10-29) in the lean group and 11 (10-16) in the obese group (P=0.20). The extent of global glomerulosclerosis was identical in the lean and obese groups, 0% (0-4.3%) and 0% (0-4.5%), respectively (P=0.89). These globally sclerotic glomeruli were not included in the morphometric measurements. The extent of segmental glomerulosclerosis was similar in both groups, 0% (0-4.4%) in the lean group and 0% (0-4.5%) in the obese one (P=0.94). The median number of non-globally sclerotic glomeruli was 19 (8-28) in the lean and 10 (9-14) in the obese groups (P=0.32). The number of non-globally sclerotic glomerular sections analyzed was 266 and 133 in the lean and obese groups, respectively. The number of proximal tubular sections analyzed was 210 and 165 in the lean and obese groups, respectively. Table 2 shows that the glomerular tuft cross-sectional area and volume in the obese group were 61% and 104% higher, respectively, than in the lean group. The proximal tubular epithelium’s cross-sectional area was 33% higher in the obese group. Table 3 shows that Bowman’s space cross-sectional area and volume in the obese group were 41% and 94% higher, respectively, than in the lean group. The cross-sectional area of the proximal tubular lumen was 54% higher in the obese group. Figure 1 and Figure 2 show representative sections of the glomerular tuft, Bowman’s space, proximal tubular epithelium and proximal tubular lumen in the lean and obese groups.

**Table 2:** Glomerular tuft cross sectional area, glomerular tuft volume and proximal tubular epithelium cross-sectional area in obese and lean groups.

	**Lean group**	**Obese group**	**P**
**Glomerular tuft cross-sectional area** (**x 10^3^ μ^2^**)	15.8 (13.6-18.2)	25.5 (21.3-31.7)	0.001
**Glomerular tuft volume (x10^6^ μ^3^**)	2.5 (2.0-3.1)	5.1 (3.9-7.1)	0.001
**Proximal tubular epithelium cross-sectional area (x 10^3^ μ^2^**)	2.98±0.98	3.95±0.93	0.02

**Table 3:** Bowman’s space cross sectional area, Bowman’s space volume and proximal tubular lumen cross-sectional area in obese and lean groups.

	**Lean group**	**Obese group**	**P**
**Bowman’s space cross-sectional area (x 10^3^ μ^2^**)	3.4±1.8	4.8±1.6	0.049
**Bowman’s space volume (x10^6^ μ^3^**)	0.83±0.47	1.61±0.70	0.003
**Proximal tubular lumen cross-sectional area** (**x 10^3^ μ^2^**)	966±417	1484±552	0.01

**Figure 3:**
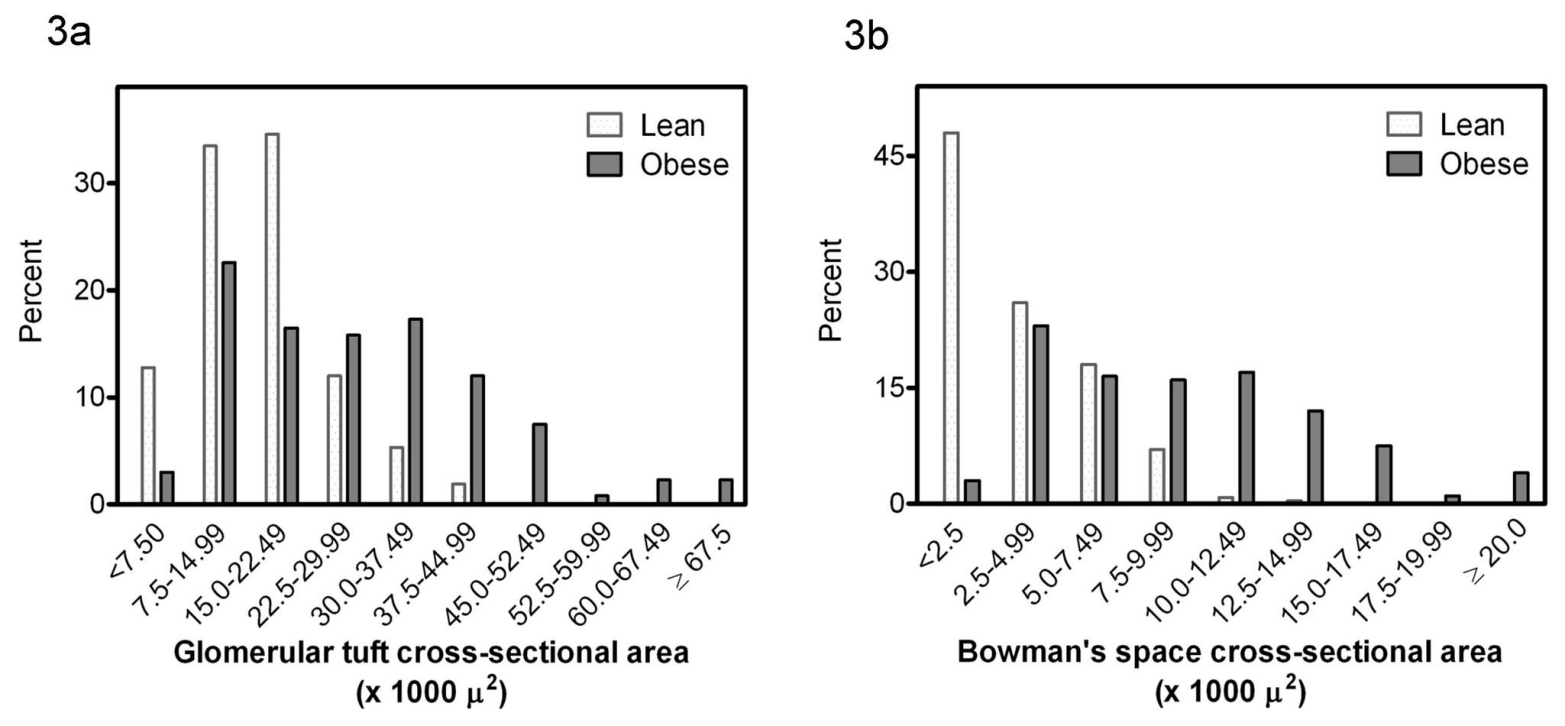
Distribution frequency of the glomerular tuft cross-sectional area (a) and Bowman’s space cross-sectional area (b) in lean and obese subjects.

**Figure 4:**
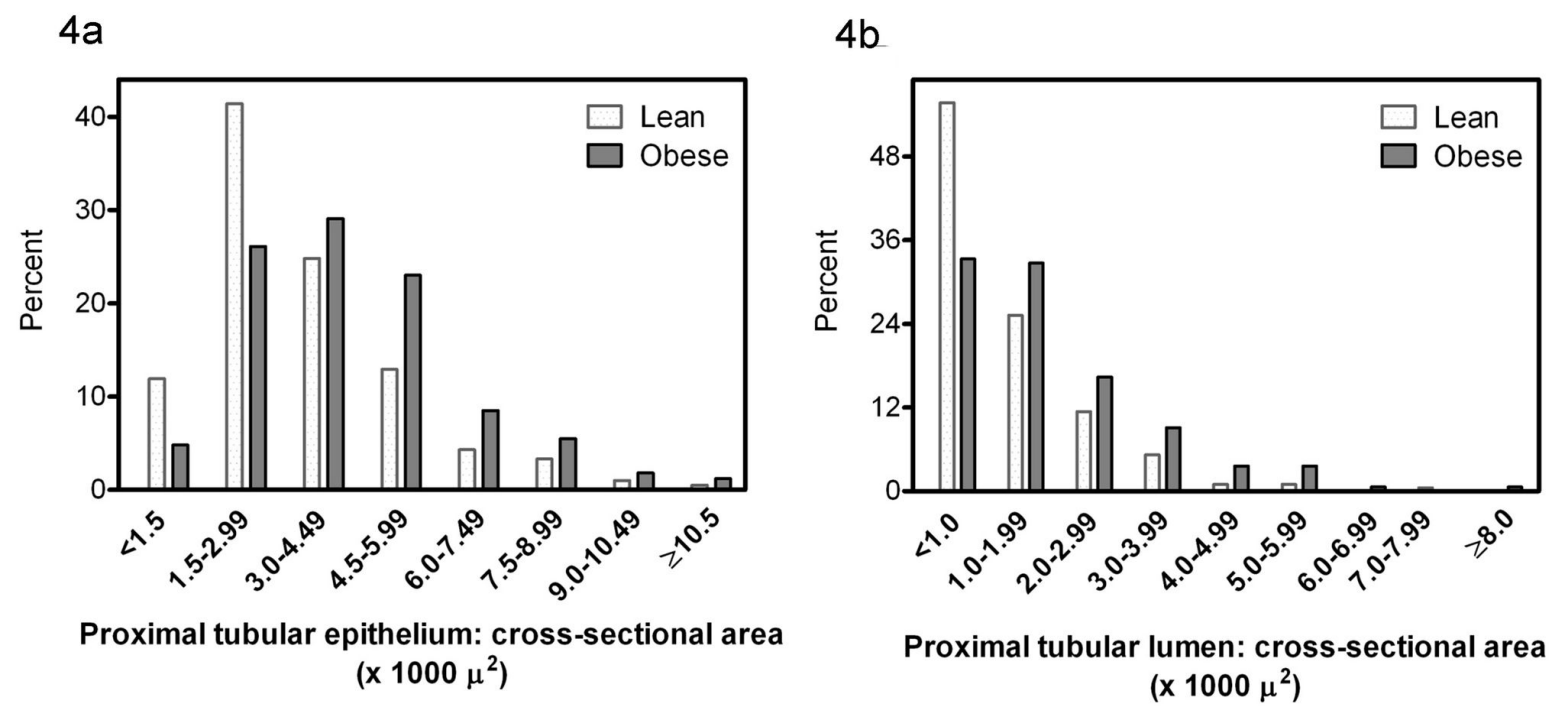
Distribution frequency of the cross-sectional area of the proximal tubular epithelium (a) and of the cross-sectional area of the proximal tubular lumen (b) in lean and obese subjects.

The distribution frequency of the glomerular tuft cross-sectional area and Bowman’s space cross-sectional area in the two groups is depicted in Figure 3. The distribution frequency of the proximal tubular epithelial and luminal cross-sectional areas is depicted in Figure 4. The distribution of these four variables is shifted to the right in the obese as compared to the lean group, i.e. toward the higher cross sectional area categories.

The number of nuclei per proximal tubular profile was 11.4±2.2 and 12.6±3.6 in the lean and obese groups, respectively (P=0.29), suggesting that the increase in tubular size was not due to tubular cell hyperplasia.

## Discussion

This study demonstrates that the volume of the proximal tubule of non-diabetic obese subjects with glomerular hyperfiltration and proteinuria is increased, owing to an increase in both proximal tubular epithelial and luminal volume. This finding is novel and has not been demonstrated either in humans or in obese animals. The increase in tubular epithelial volume is accounted for by hypertrophy and not by hyperplasia. This increase in proximal tubular volume is associated with a two-fold increase in glomerular tuft volume, in agreement with previous studies, and with a similar increase in Bowman space volume, not previously reported in obese humans.

The obese and lean groups studied were similar as far as height, gender, proteinuria and serum albumin concentrations were concerned. Glomerular filtration rate was normal in the lean group and increased by 55% in the obese group. Proteinuria was similar in both groups. The degree of chronicity of the kidney biopsy was mild in both groups. Diastolic arterial pressure was normal and similar in both groups. The mean systolic arterial pressure, although normal in both groups, was higher in the obese than in the lean group. The obese group was older. Considering that the expected effect of ageing on tubular and glomerular volumes is a decline, the age factor cannot account for the higher glomerular and tubular volumes revealed in the obese group. The present study revealed that obese subjects had a larger glomerular tuft area than the lean subjects, consistent with previous findings in human studies [1,21-30]. This 61% increase in tuft area and doubling of glomerular tuft volume was associated with a 50% increase in glomerular filtration rate, confirming that glomerulomegaly is associated with glomerular hyperfiltration. Bowman’s space area was twice that of the lean group, a finding not previously reported in obese humans. The present study demonstrates for the first time that glomerulomegaly in obesity is associated with an increase in proximal tubular epithelial and luminal volume.

What are the implications of these morphological findings? Adiposity is associated with increased glomerular filtration rate [6,8,10,11,13,14] and proximal fractional reabsorption of sodium [13,35]. How are the structural findings reported in the present study relevant to the functional changes affecting the kidney in obesity?

### Tubular epithelial hypertrophy

As a direct consequence of glomerular hyperfiltration, regardless of its cause – obesity or diabetes mellitus, the sodium filtered load is increased. The enhanced sodium load has to be reabsorbed along the nephron in order to avoid salt wasting. Experimental and clinical investigations revealed that proximal reabsorption is increased in murine and human diabetes mellitus [38-40] and in obese non-diabetic subjects [13,35]. Vallon, Blantz and Thomson [41] presented data obtained in a murine diabetic model suggesting that proximal tubular growth is necessary in order to allow enhanced proximal tubular reabsorption, leading to decreased solute delivery to the macula densa and deactivation of the tubuloglomerular feedback, with consequent glomerular hyperfiltration. Thus, the presence of tubular hypertrophy in obese subjects is important since it represents the structural basis facilitating tubular hyperfunction. In addition, tubular hypertrophy may be important as an epiphenomenon. Studies in diabetic subjects showed that renal hypertrophy, i.e. tubular hypertrophy, is a risk factor for the development of chronic kidney disease, independently of albuminuria or hyperfiltration [42-44]. Growth factors involved in cell hypertrophy play a role in different models of chronic kidney disease through activation of proinflammatory and profibrotic factors [45,46].

### Increased glomerular tuft and Bowman’s space volume

Besides the known increase in glomerular tuft volume [1,21-30], the present study provides the first evidence that Bowman’s space is increased in obese subjects. A single experimental study revealed an increased Bowman’s space in obese dogs [31]. In that model, glomerular hyperfiltration was also associated with increased glomerular volume; however, glomerulomegaly was mostly accounted for by an increase in Bowman’s space volume and not by an increase in glomerular tuft volume.

The association between glomerular hyperfiltration and increased Bowman’s space suggests that urinary space dilation may be the consequence of the high hydrostatic pressure gradient across glomerular capillaries [10]. The resultant elevated glomerular ultrafiltration rate is expected to lead to increased hydrostatic pressure in Bowman’s space and to its consequent dilation. This dilation is expected to result in a decrease in Bowman’s space pressure, allowing maintenance of a high transcapillary hydrostatic pressure gradient and perpetuation of glomerular hyperfiltration. We speculate that in the absence of Bowman’s space dilation, the high Bowman’s space hydrostatic pressure would lead to damage to the parietal epithelial cells, triggering formation of synechiae and segmental sclerosis. The increased Bowman’s space pressure would also cause a drop in glomerular transcapillary hydrostatic pressure gradient and in a consequent decrease in the glomerular ultrafiltration rate with ensuing increase in glomerular hydrostatic capillary pressure and damage to endocapillary structures. Thus Bowman’s space dilation should be considered as protective, attenuating high pressure mediated glomerular injury. The fact that the proximal tubular lumen volume is also increased suggests that Bowman’s space dilation was insufficient to normalize Bowman’s space pressure and that the increased pressure was transmitted distally.

Glomerular tuft hypertrophy may be in part responsible for the podocytes’ abnormalities described in obesity-related glomerulopathy [25,47]. Nagata and Kriz [48] showed that glomerular growth triggers maladaptive structural changes, eventually leading to podocyte depletion, adherence of capillaries to parietal epithelium and segmental glomerulosclerosis. In addition to the damage induced by this growth-related mechanical stress, the increase in Bowman’s space pressure may directly affect glomerular epithelial cells. Endlich et al [49] provided evidence that podocytes respond in vitro to mechanical stress, resulting in morphologic and cytoskeletal changes. Mechanical stress may alter podocytes by activating local angiotensin and TGF_β_ pathways systems [50].

### Increased tubular urinary space

The presence of proximal tubular urinary space dilation indicates past or present luminal hypertension in the proximal tubule. It should be considered as a mechanism that off-sets the deleterious glomerular and tubulo-interstitial consequences of obesity-related glomerular hyperfiltration. In addition, an increased pressure in the proximal tubular lumen may play a role in the pathogenesis of interstitial fibrosis. Proximal tubular cells are mechanosensitive [51]. Sonomura et al [52] recently demonstrated that mechanical stress on renal tubules may increase the expression of pro fibrotic factors.

It should be emphasized that this investigation was performed in obese subjects with proteinuria. Most of these subjects had obesity-related glomerulopathy. These findings should not be generalized to the obese population without proteinuria.

In conclusion, obesity-related glomerular hyperfiltration is associated with proximal tubular epithelial hypertrophy and increased glomerular and tubular urinary space volume in subjects with proteinuria; the expanded glomerular and urinary space is probably a direct consequence of glomerular hyperfiltration. These effects may be involved in the pathogenesis of obesity-related renal disease.
